# Comprehensive Treatment of Trans-Arterial Chemoembolization Plus Lenvatinib Followed by Camrelizumab for Advanced Hepatocellular Carcinoma Patients

**DOI:** 10.3389/fphar.2021.709060

**Published:** 2021-10-18

**Authors:** Juanfang Liu, Zhen Li, Wenguang Zhang, Huibin Lu, Zhanguo Sun, Guozheng Wang, Xinwei Han

**Affiliations:** Department of Interventional Radiology, The First Affiliated Hospital of Zhengzhou University, Zhengzhou, China

**Keywords:** hepatocellular carcinoma, TACE, lenvatinib, camrelizumab, intervention therapy

## Abstract

**Aim:** This study aimed to report the efficacy and safety of *trans*-arterial chemoembolization (TACE) plus lenvatinib and camrelizumab in patients with advanced hepatocellular carcinoma (HCC).

**Methods:** This retrospective study enrolled 22 patients with advanced HCC from March 2018 to December 2019. All the patients received comprehensive treatment with TACE plus lenvatinib followed by camrelizumab. Overall survival (OS) and progression-free survival (PFS) were calculated and analysed using the Kaplan-Meier method and log-rank test. Treatment response and adverse events (AEs) were also evaluated.

**Results:** The objective response rate (ORR) and disease control rate (DCR) for the whole cohort were 68.2 and 100% at the first month and 72.7 and 95.5% at the third month, respectively. The median OS was 24 months (95% CI, 20.323–27.677 months), and the median PFS was 11.4 months (95% CI, 8.846–13.954 months). The majority of treatment-related adverse reactions were mild or moderate, except for 4 that developed to grade 3–4 (3 reactions of grade 3, 1 reaction of grade 4). No deaths or other serious adverse reactions occurred.

**Conclusion:** *Trans*-arterial chemoembolization plus lenvatinib and camrelizumab shows good results incontrolling tumour progression and prolonging median OS in patients with advanced HCC.

## Introduction

Hepatocellular carcinoma (HCC) is one of the leading causes of cancer-related death worldwide (Ferlay et al., 2015). It represents approximately 90% of primary liver cancers and constitutes a major global health problem (Galle Peter et al., 2018). Due to its insidious onset and nonspecific symptoms, HCC is often diagnosed at a late or advanced stage. According to the EASL and AASLD guidelines, TACE is recommended as the standard therapeutic method for intermediate HCC of BCLC stage B; for late stage patients, systemic treatment is recommended (Galle Peter et al., 2018; Heimbach et al., 2018). However, in certain circumstances, the indications for TACE have been extended for CNLC stages IIb, IIIa, and some IIIb HCCs (Xie et al., 2019).

Lenvatinib is an oral tyrosine kinase inhibitor that plays an anticancer role by effectively inhibiting vascular endothelial growth factor receptor 1–3 (VEGFR1–3), platelet-derived growth factor receptor α, RET proto-oncogene (RET), and v-kit Hardy-Zuckerman 4 (KIT) (Yamamoto et al., 2014). Lenvatinib has been approved for first-line treatment of patients with unresectable advanced HCC in many countries. The safety and efficacy of lenvatinib for HCC patients has been demonstrated in clinical practice in many studies (Chuma et al., 2020; Han et al., 2020). Since 2018, lenvatinib has become another first-line systemic treatment for HCC patients and is deemed an alternative to sorafenib due to its comparable efficacy (Kudo et al., 2018).

Recently, antibodies targeting the programmed cell death-1 (PD-1) pathway have been widely used in numerous combination regimens to improve the tumour response and prolong the overall survival (OS) of HCC patients. In 2019, the domestic anti-PD-1 antibody camrelizumab was first approved in China and subsequently applied for various malignancies, including B cell lymphoma, oesophageal squamous cell carcinoma and hepatocellular carcinoma (Markham and Keam, 2019). The extensively common combination therapy for advanced HCC comprises anti-PD1 plus tyrosine kinase inhibitor agents. A multicentre phase 2 trial conducted by Xu J and his colleagues found that the combination therapy of camrelizumab plus apatinib in advanced HCC patients resulted in median progression-free survival in two cohorts of 5.7 and 5.5 months, and the associated 12 months survival rates were 74.7 and 68.2%. Recently, Yuan G et al. showed that camrelizumab plus apatinib could achieve a promising outcome in advanced HCC patients with portal vein tumour thrombus (PVTT) (Yuan et al., 2020). There were also many studies showed encouraging antitumor activity and acceptable toxicity in patients with advanced non-small-cell lung cancer (NSCLC) and esophageal squamous cell carcinoma treated with camrelizumab plus apatinib (Yan et al., 2020; Zhou et al., 2021).

Repeated *trans*-arterial chemoembolization (TACE) is associated with a decline in liver function and poor therapeutic response. Shimose S et al. performed a propensity score matching study in 113 patients with intermediate-stage HCC, showing that alternating lenvatinib and TACE could prolong overall survival and improve prognosis (Shimose et al., 2021). Our previous study showed that TACE combined with apatinib improved OS and PFS in patients with large hepatocellular carcinoma (Liu et al., 2019). In this study, we aimed to evaluate the therapeutic efficacy of TACE plus lenvatinib and camrelizumab in advanced HCC patients.

## Materials and Methods

### Patients

Between April 2019 and December 2019, 22 patients with advanced HCC who were treated with TACE plus lenvatinib followed by camrelizumab were enrolled in this retrospective study. All patients were followed-up until death or the last ambulatory visit until February 28, 2021. The inclusion criteria were 1) a diagnosis of HCC according to EASL or AASLD; 2) age ≥18 years; 3) BCLC stage B or C and inability to tolerate or refusal of surgery, radiation or ablation; 4) Child-Pugh class A or B; and 5) absence of heart, lung or kidney dysfunction. The exclusion criteria were 1) severe coagulation disorders; 2) refractory ascites; 3) an expected survival time of less than 3 months; and 4) treatment discontinuation due to severe adverse reactions or abandonment. This study conformed to the rules of the Declaration of Helsinki and was approved by the institutional review board of Zhengzhou University First Affiliated Hospital.

### Procedures

#### TACE Procedure

The TACE procedure was consistent with that described by Lu W (Lu et al., 2017). Conventional angiography of the proper hepatic artery, superior mesenteric artery, phrenic arteries and left gastric artery was performed to determine the blood supply to the tumours. Then, 4 mg of raltitrexed diluent and 100 mg of oxaliplatin were slowly injected through the *trans*-arterial catheter. Next, emulsions of 10–20 ml of lipiodol and 20 mg pirarubicin were infused via the microcatheter into the feeding arteries of the tumours. Finally, the feeding arteries were embolized with gelfoam particles. Considering the late stage of HCC, all of the patients underwent the TACE procedure only approximately 1–3 times based on imaging examination findings, and liver function was rechecked during follow-up.

### Lenvatinib Treatment Protocol

Lenvatinib (Eisai Co., Ltd., Tokyo, Japan) was orally administered at a dose of 12 mg/day for patients with body weight *≥*60 kg or 8 mg/day for patients with body weight <60 kg in accordance with the manufacturers’ instructions. Drug dose reduction or interruption was permitted until severe adverse events occurred.

### Camrelizumab Doses

Camrelizumab (Jiangsu Hengrui Medicine Co. Ltd., Jiangsu, China) was given at a fixed dose of 200 mg every 3 weeks (q3w) intravenously. Camrelizumab administration continued until intolerable toxicity or disease progression occurred. Dose interruption for no more than 12 weeks was permitted.

### Clinical and Laboratory Evaluation

Tumour response was evaluated based on enhanced CT or MRI findings according to the modified Response Evaluation Criteria in Solid Tumors (mRECIST) (Seyal et al., 2015), including complete response (CR), partial response (PR), standard deviation (SD), and progressive disease (PD). Laboratory data, including alanine aminotransferase (ALT), aspartate aminotransferase (AST), albumin (ALB) and total bilirubin (TBIL), were collected to evaluate liver function before treatment (D0), 1 week after the first cycle of operation (D7) and 1 month after the first cycle of treatment (D30). Treatment-related adverse events (TRAEs) were assessed according to the National Cancer Institute (NCI) Common Terminology Criteria for Adverse Events (CTCAE v4.03). Efficacy of treatment was measured by overall survival (OS) and progression-free survival (PFS), which were defined as the time between first combination therapy to death or the last follow-up and the time between first therapy to tumour progression or death, respectively.

### Statistical Analysis

All data were analyzed using the statistical software SPSS 19.0 (SPSS Inc., Chicago, IL, United States). Data are expressed as percentages for categorical variables and as the mean ± standard deviation (SD) or median (25th–75th percentiles) for continuous variables. Comparisons between two groups pre- and post-treatment were assessed by the paired *t* test. Survival curves were calculated by using the Kaplan-Meier method. Univariable analyses were performed with the log-rank test. Kaplan–Meier survival curves were used to examine PFS and OS. Univariable and multivariable Cox proportional hazards regression analyses were used to predict prognostic factors of PFS and OS. A *p*-value < 0.05 was considered statistically significant.

## Results

### Patient Characteristics

The mean age of the patients was 57.7 ± 9.9 years. Of the 22 patients, 17 (77.3%) were men and predominantly had hepatitis B virus infection (68.2%). Among all patients, 16 (72.7%) patients had Child–Pugh A HCC and 6 (27.3%) had Child–Pugh B HCC. The numbers of patients with BCLC stages B and C HCC were 12 (54.5%) and 10 (45.5%), respectively. Eleven patients (50%) were considered to have portal vein tumour thrombus (PVTT). Over the whole therapy cycle, only 3 (13.6%) patients had a reduction in lenvatinib, and 1 (4.5%) patient had a temporary suspension of camrelizumab as a result of not being well tolerated. Detailed characteristics of the patients are listed in Table 1.

**TABLE 1:** Baseline characteristics.

Parameters	Value
Age (years)	57.7 ± 9.9
Sex (n/%)	
Male	17 (77.3)
Female	5 (23.7)
Cause of cirrosis (n/%)	
Hepatitis B	15 (68.2)
Hepatitis C	4 (18.2)
Others	3 (13.6)
ECOG performance status (n/%)	
0–1	14(63.6)
2	8 (36.4)
BCLC stage (n/%)	
B	12 (54.5)
C	10 (45.5)
Child-Pugh class (n/%)	
A	16 (72.7)
B	6 (27.3)
PVTT (n/%)	11 (50.0)
Intrahepatic metastasis (n/%)	8 (36.4)
Tumour burden (n/%)	
≤50%	14 (63.6)
>50%	8 (36.4)
Tumour location (n/%)	
Left	5 (23.7)
Right	13 (59.1)
Both	4 (18.2)
AFP (>400 ng/ml) (n/%)	15 (68.2)

PVTT: portal vein tumour thrombus; ECOG: Eastern Cooperative Oncology Group; BCLC: Barcelona Clinic Liver Cancer.

### Safety

**FIGURE 1:**
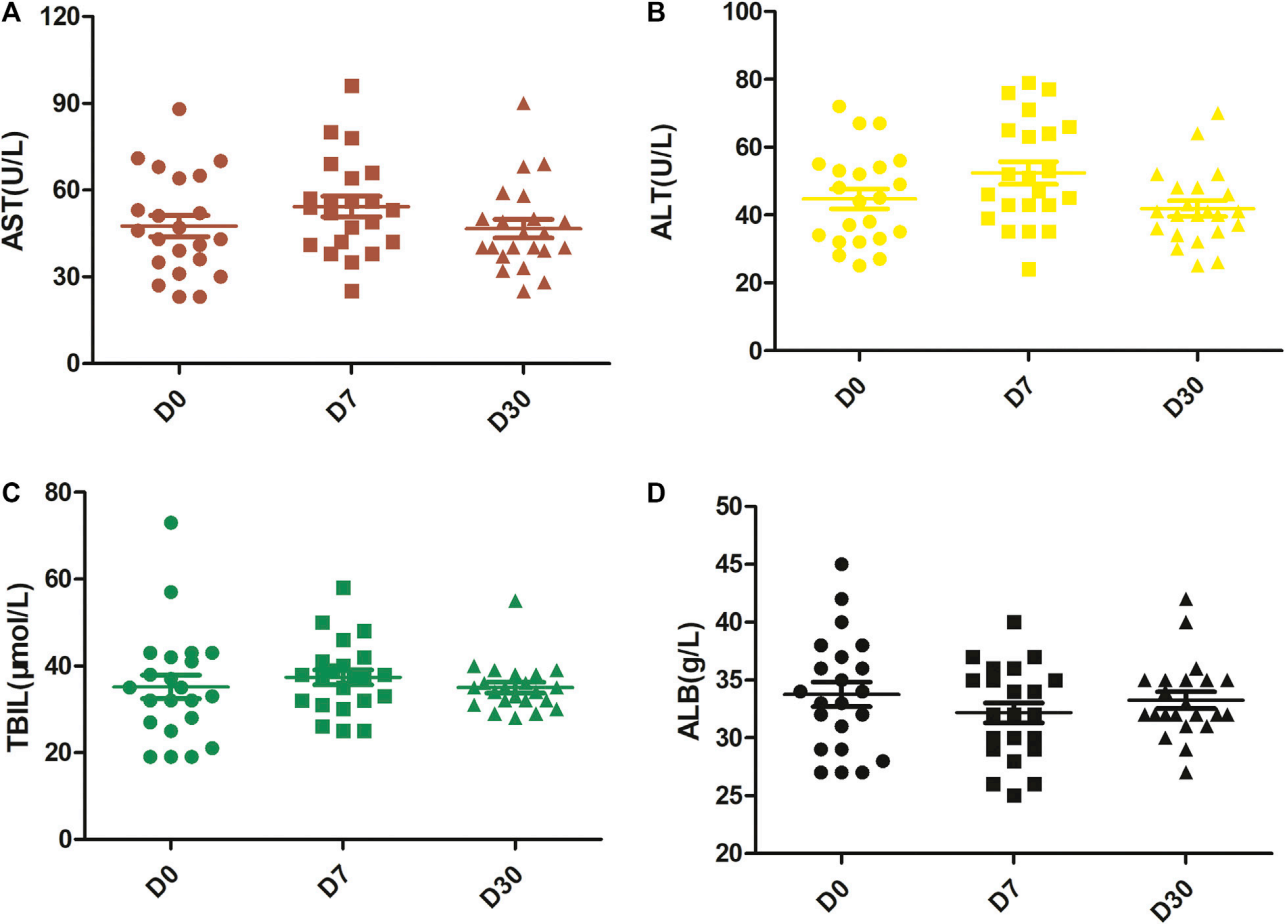
Changes in liver function over time. The levels of AST **(A)** and ALT **(B)** were significantly enhanced at D7 with respect to D0, but they returned to baseline levels at D30. There were no significant differences in the levels of TBIL **(C)**. The level of ALB **(D)** was reduced at D7 but returned to the baseline level at D30. D0, pretreatment; D7, 1 week after the first treatment cycle; D30, 1 month after the first treatment cycle.

One week after combination therapy (D7), the levels of AST and ALT were elevated (54.27 ± 16.55 vs. 47.55 ± 17.46, *p* < 0.001; 52.36 ± 15.57 vs. 44.68 ± 13.74, *p* < 0.001, respectively), and the level of ALB was reduced (32.18 ± 4.07 vs. 33.77 ± 4.98, *p* = 0.011) compared with that prior to treatment (D0). However, there was no difference in TBIL on D7 vs. D0 (*p* = 0.154) or on D30 vs. D0 (*p* = 0.921). All of these levels returned to baseline levels 1 month after the first cycle of combination therapy (D30). Thus, no differences were observed in the levels of AST, ALT, ALB and TBIL between D30 and D0 (all *p* > 0.05) (Figure 1).

### Efficacy

**FIGURE 2:**
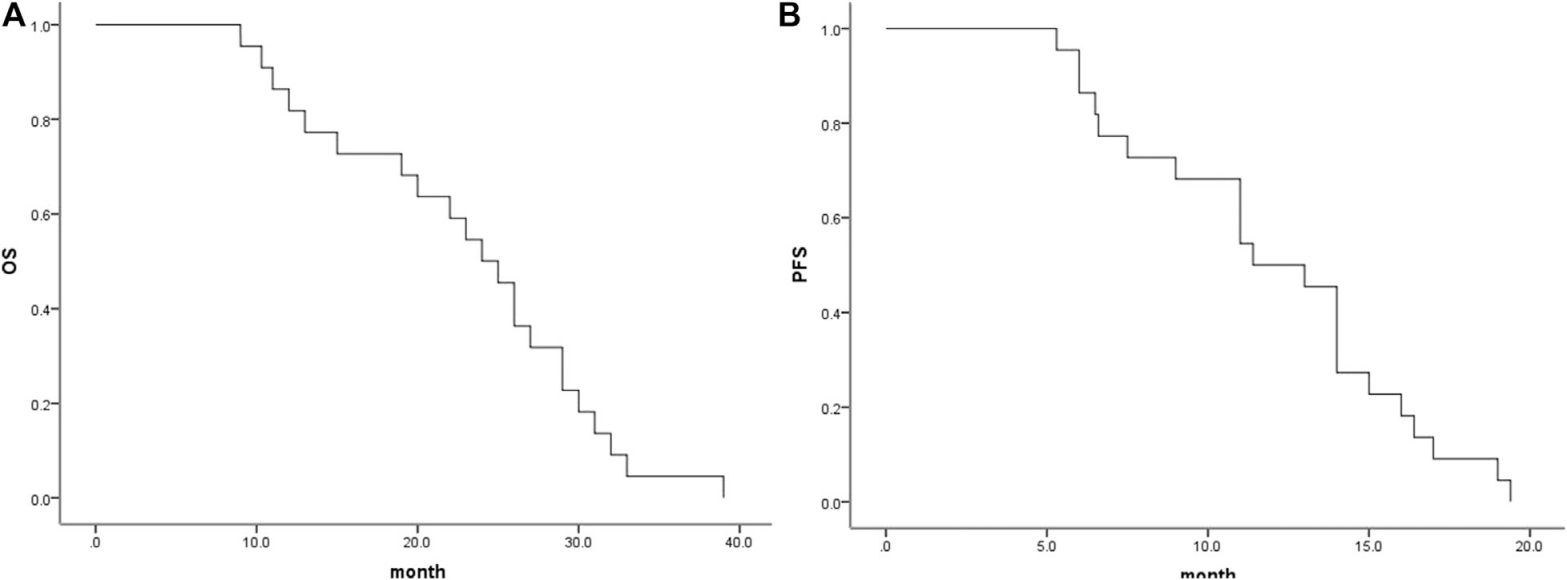
Median OS and PFS for all patients. **(A)**, Graph indicates a median OS of 24.0 months (95% CI, 20.32–27.67 months); **(B)**, Graph indicates a median PFS of 11.4 months (95% CI, 8.85–13.95 months). OS, overall survival; PFS, progression-free survival.

In the analysis of tumour response according to the first follow-up CT and MRI 1 and 3 months after combination therapy, the ORRs were 96 and 94%, respectively, and the DCRs were 100 and 96%, respectively (Table 2). The Kaplan-Meier curves for OS and PFS are shown in Figure 2. The median PFS was 9.5 months (95% CI, 8.1–10.9 months), and the median OS was 22.0 months (95% CI, 20.2–23.9 months). The 1- and 2-years OS rates were 62.5 and 20.5%, respectively.

**TABLE 2:** Treatment responses at different times.

	CR	PR	SD	PD	ORR	DCR
M1	0	15	7	0	68.2	100
M3	0	16	5	1	72.7	95.5

M1: first month ; M3: ; CR: complete response; PR: partial response; SD: standard deviation; PD: progressive disease; = CR + PR; = CR + PR + SD.

### Adverse Events

All recorded treatment-related adverse events (TRAEs) are shown in Table 3. Fourteen patients (63.6%) experienced at least one TRAE. Overall, the most common TRAEs were nausea (45.5%), abdominal pain (36.4%), fever (36.4%), hepatic function abnormalities (23.7%), bone marrow suppression (18.2%), hand-foot skin reactions (18.2%), fatigue (23.7%) and hypertension (23.7%). For all included patients, TRAEs were mild to moderate, and no toxicity-induced death occurred in this study. Grade 3/4 TRAEs occurred in four patients (18.2%): two people had hypertension, 1 had hand-foot syndrome and was forced to have a reduced dose of lenvatinib, and 1 had grade 4 dysphonia that finally led to treatment interruption of camrelizumab after 6 months of therapy.

**TABLE 3:** Adverse reactions.

Adverse reactions			
	G1-2	G3	G4
Abdominal pain	8 (36.4)	—	—
Fever	8 (36.4)	—	—
Nausea	10 (45.5)	—	—
Vomiting	7 (31.8)	—	—
Bone marrow suppression	4 (18.2)	—	—
Hepatic function abnormal	5 (23.7)	—	—
Arthralgia	3 (13.6)	—	—
Fatigue	5 (23.7)	—	—
Hypertension	5 (23.7)	2 (9.1)	—
Hand-foot syndrome	4 (18.2)	1 (4.5)	—
Diarrhoea	2 (9.1)	—	—
Anorexia	2 (9.1)	—	—
Proteinuria	3 (13.6)	—	—
Rash	3 (13.6)	—	—
Stomatitis	1 (4.5)	—	—
Gingival bleeding	2 (9.1)	—	—
Dysphonia	1 (4.5)	—	1 (4.5)

### Factors Affecting OS and PFS

Univariable Cox proportional hazard regression analysis indicated that ECOG performance status (2 vs. 0–1) was correlated with both shorter OS and shorter PFS (*p* < 0.001 and *p* = 0.0015, respectively). Additionally, Child-Pugh class (A vs. B) (*p* = 0.001 and 0.022, respectively), PVTT (*p* = 0.002 and *p* = 0.023, respectively) and extrahepatic metastasis (both *p* < 0.005) were associated with shorter OS and PFS. Intrahepatic metastasis was related to a shorter OS (*p* = 0.030) but not a shorter PFS (*p* = 0.333). Multivariable Cox regression revealed that PVTT (*p* = 0.024) and extrahepatic metastasis (*p* = 0.039) independently predicted worse OS in patients with advanced HCC and that extrahepatic metastasis (*p* = 0.022) independently predicted shorter PFS in patients with advanced HCC (Table 4).

**TABLE 4:** Factors affecting OS and PFS.

Parameters	OS				PFS			
	HR	95% CI		p	HR	95% CI		p
		lower	higher			lower	higher	
Univariable Cox regression								
Age (60 vs. 60y)	1.176	0.483	2.862	0.721	0.904	0.377	2.168	0.820
BCLC (C vs. B)	1.870	0.769	4.546	0.167	1.440	0.599	3.460	0.415
Sex (Male vs. Female)	3.036	0.942	9.778	0.063	1.870	0.642	5.448	0.251
ECOG performance status (2 vs. 0–1)	12.076	3.496	41.710	<0.001	4.151	1.830	9.416	0.001
Child-Pugh class (B vs. A)	10.580	2.566	42.628	0.001	3.262	1.183	9.000	0.022
PVTT	7.623	2.076	27.990	0.002	3.219	1.171	8.851	0.023
Intrahepatic metastasis	2.952	1.110	7.848	0.030	1.560	0.634	3.838	0.333
Extrahepatic metastasis	15.629	3.532	69.147	<0.001	8.858	2.441	32.139	0.001
Multivariable Cox regression								
Child-Pugh class (B vs. A)	1.360	0.186	9.938	0.762	0.861	0.149	4.982	0.867
ECOG performance status (2 vs. 0–1)	2.592	0.852	7.888	0.094	1.666	0.580	4.782	0.343
Intrahepatic metastasis	1.418	0.438	4.595	0.560	0.747	0.232	2.410	0.625
PVTT	5.555	1.259	24.503	0.024	2.969	0.844	10.445	0.090
Extrahepatic metastasis	7.336	1.108	48.562	0.039	7.932	1.351	46.573	0.022

OS: overall survival; HR: hazard ratio; CI: confidence interval; PFS: progression-free survival.

## Discussion

Currently, *trans*-arterial chemoembolization (TACE) is generally considered the first-line therapy for intermediate-advanced HCC patients (Covey and Hussain, 2017; Wu et al., 2018). However, the local hypoxic environment induced by embolization is a dangerous factor for tumour recurrence and can stimulate neovascularization in the tissue surrounding the tumours. Recently, a small-molecule inhibitor of VEGFR1-3, lenvatinib, was approved for the first-line treatment of patients with advanced HCC in many countries and was shown to be superior to sorafenib (Al-Salama et al., 2019). Many studies have demonstrated that the combination of TACE and VEGF inhibitors shows good results in advanced HCC (Jayson et al., 2016; Piñero et al., 2019). Importantly, recent results of a phase II/III clinical trial showed that camrelizumab was effective for advanced HCC, with an ORR of 13.8% and a 6 months OS rate of 74.7% (Markham and Keam, 2019). Additionally, combination therapy with VEGF inhibitors and PD-1-targeted immunotherapy has been recommended as the standard of therapy for patients with advanced HCC (Cersosimo, 2020). In our study, we found that combination therapy of TACE, lenvatinib and camrelizumab for HCC patients was efficacious: the ORRs at M1 and M3 were 68.2 and 72.7%, respectively. In addition, the DCR reached 100 and 95.5% at M1 and M3, respectively. In the present study, the median OS was slightly longer than that for TACE combined with apatinib revealed in our prior study (24.0 vs. 22 months). An obvious survival benefit could be seen in our study, with a median OS of 24 months when compared with the study reported by Chen S et al., who found an OS of 13 months using a combination therapy of TACE plus apatinib (Chen et al., 2018). We also found that the median OS was longer than that of TACE combined with sorafenib as reported by Pawlik T et al. in advanced HCC patients (Pawlik et al., 2011). The main reason might be that lenvatinib plus an immune checkpoint inhibitor (camrelizumab) enhanced the anticancer efficacy, as lenvatinib could alleviate hypoxia and remodel the immunosuppressive tumour environment. Additionally, the median OS in our study was obviously longer than that reported by Yuan G, who conducted a multicentre retrospective study and found that the median OS was 14.8 months in patients with PVTT treated with camrelizumab combined with apatinib (Yuan et al., 2020). These could be due to that: 1) DEB-TACE are developed for loading and slowly releasing cytotoxic drugs into the tumour, and they also act as embolization agents to block blood supply to hypervascular tumors and thereby more effectively killing cancer cells and inducing tumor necrosis compared with non-TACE. Thus, more favorited survival profiles were observed in our study.

In this study, univariable Cox regression analysis indicated that there was no significant correlation of median PFS or median OS with sex or age. Multivariable Cox regression analysis revealed that extrahepatic metastasis independently predicted a shorter OS and PFS in patients with advanced HCC.

Embolic syndrome induced by TACE and the common adverse events of lenvatinib and camrelizumab might cause liver dysfunction, pain, ascites, diarrhoea, anorexia and proteinuria. In this study, the levels of AST and ALT at D7 were elevated but returned to baseline levels 1 month (D30) after the first cycle of combination therapy. No severe liver dysfunction occurred. During the whole study, grade 3/4 AEs occurred in 3 (13.6%) patients: two people had hypertension and were forced to have their lenvatinib doses reduced, and 1 had grade 3 dysphonia that finally led to treatment interruption of camrelizumab after 6 months of therapy. The remaining patients all tolerated their adverse reactions. Overall, the adverse reactions among the patients were acceptable, and no serious adverse reactions occurred.

This study was a retrospective observational study; thus, several major limitations were present. First, the number of patients in our study was small, and the follow-up time was relatively short. Second, the study did not use a case-control design. Large-scale multicentre prospective studies are required to verify the effect and safety of TACE combined with lenvatinib and camrelizumab for advanced HCC patients.

Despite these limitations existed, the results showed that TACE combined with lenvatinib and camrelizumab could prolong the median OS and PFS without serious adverse reactions in advanced HCC patients. This method may provide another form of treatment for the comprehensive management of late-stage HCC.

## Data Availability

The raw data supporting the conclusion of this article will be made available by the authors, without undue reservation.
